# Usefulness of intraoperative neuromonitoring for preservation of an extralaryngeal bifurcation of the recurrent laryngeal nerve: A case report

**DOI:** 10.1016/j.ijscr.2018.11.009

**Published:** 2018-11-14

**Authors:** Masatsgu Yano, Yasufumi Saito, Makoto Yoshida, Takafumi Oshiro, Toshikatsu Fukda, Makoto Ochi, Yuzo Okamoto, Eiji Ono, Hideki Ohdan

**Affiliations:** a Department of Surgery, JR Hiroshima Hospital, Hiroshima, Japan; b Department of Gastroenterological and Transplant Surgery, Graduate School of Biomedical and Health Sciences, Hiroshima University, Hiroshima, Japan

**Keywords:** Extralaryngeal bifurcation, Recurrent laryngeal nerve, Thyroid surgery, Neuromonitoring

## Abstract

Introduction: Recurrent laryngeal nerve injury is a major complication of thyroid surgery. An endotracheal tube with electromyography electrodes attached to it was recently developed for intraoperative neuromonitoring during thyroid surgery. Here we describe the successful identification and preservation of an extralaryngeal bifurcation of the recurrent laryngeal nerve by intraoperative neuromonitoring in a patient undergoing thyroid surgery.

Presentation of case: A 56-year-old woman presented for evaluation of a neck swelling found during a medical examination. Computed tomography (CT) revealed a tumor with a 5-cm diameter in the left thyroid lobe. Fine needle aspiration cytology revealed a Bethesda category III finding. Left thyroid lobe resection was scheduled. During surgery, the left recurrent laryngeal nerve was found to be adhered to the tumor. Careful exploration and intraoperative neuromonitoring allowed us to identify and preserve an extralaryngeal bifurcation of the recurrent laryngeal nerve.

Discussion: The recurrent laryngeal nerve can demonstrate various anomalies and bifurcations. Failure to notice and correctly identify extralaryngeal bifurcation leads to recurrent laryngeal nerve injury. Motor branch injury has a particularly large effect. Intraoperative neuromonitoring has been reported to be useful for identifying and preserving the recurrent laryngeal nerve and its aberrations as well as the external branch of the superior laryngeal nerve during thyroid surgery.

Conclusion: The findings from this case suggest that an extralaryngeal bifurcation of the recurrent laryngeal nerve can be identified and safely preserved by intraoperative neuromonitoring.

## Highlights

• Recurrent laryngeal nerve injury is a major complication of thyroid surgery.
• Use of an electromyography endotracheal tube can prevent this injury.
• We describe a case of extralaryngeal bifurcation of the recurrent laryngeal nerve.
• Intraoperative neuromonitoring could identify and preserve this bifurcation.

## 1 Introduction

Recurrent laryngeal nerve injury is a major complication of thyroid surgery. Oft-cited risk factors for this type of injury include Graves’ disease, large tumors with mediastinal involvement, advanced cancer, and reoperation. However, anatomical anomalies such as a nonrecurrent inferior laryngeal nerve are also major risk factors.

A recently developed electromyography endotracheal tube (NIM-Response3.0, Medtronic Japan Co. Ltd.), which is an endotracheal tube with electromyography electrodes attached to it, has been reported to be useful for identifying and preserving the recurrent laryngeal nerve and its aberrations as well as the external branch of the superior laryngeal nerve during thyroid surgery. Nerve stimulation probes electrically stimulate the nerve and the electromyography waveforms evoked from the arytenoid muscle are monitored and assessed.

Here we describe the successful identification and preservation of an extralaryngeal bifurcation of the recurrent laryngeal nerve by intraoperative neuromonitoring using an electromyography endotracheal tube.

## 2 Presentation of case

**Fig. 1:**
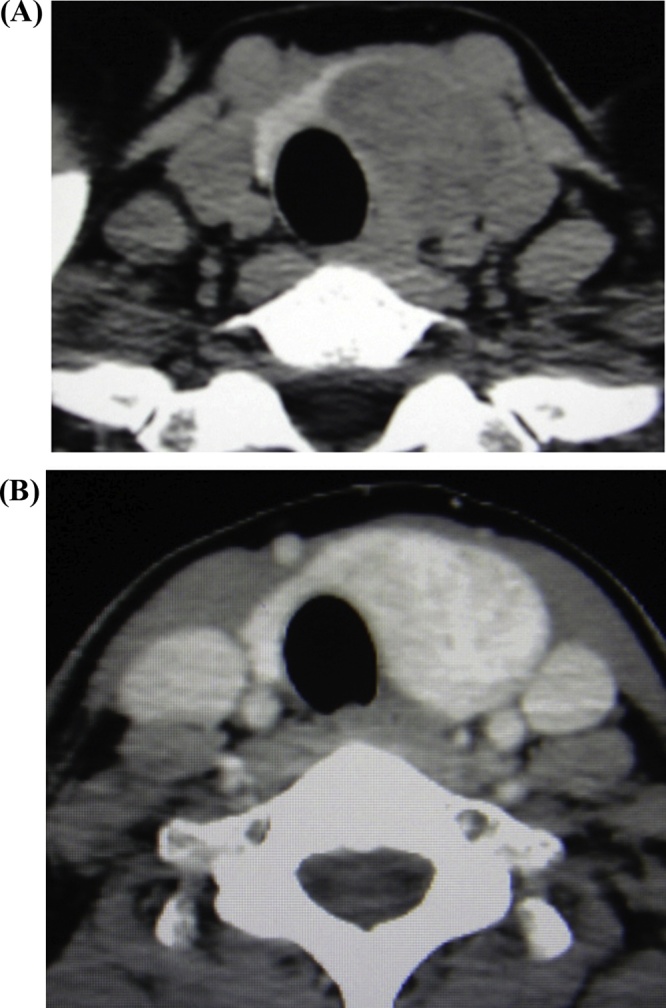
Computed tomography (CT) findings for a 56-year-old woman with a thyroid tumor. Plain CT shows a low-density tumor with a major axis of roughly 5 cm in the left thyroid lobe (A). On a contrast-enhanced CT image the internal portion of the tumor shows densely and uneven staining (B). The trachea is displaced to the right.

A 56-year-old woman presented for further evaluation of a neck swelling found during a medical examination. Her physician had performed an ultrasound examination, which revealed a tumor with an approximate diameter of 5 cm in the left thyroid lobe. She was subsequently referred to our hospital. On palpation, a mobile, elastic, soft mass with a smooth surface and a diameter of 5 cm was found in the left thyroid lobe. The cervical lymph nodes were not palpated. Ultrasound examination revealed a solid 4.8 × 3.3 cm tumor in the left thyroid lobe. Plain computed tomography (CT) showed a low-density tumor with a major axis of roughly 5 cm in the left thyroid lobe. On a contrast-enhanced CT image, the internal portion of the tumor showed dense, uneven staining. The trachea was displaced to the right (Fig. 1). Fine needle aspiration cytology revealed a Betheda category III finding.

**Fig. 2:**
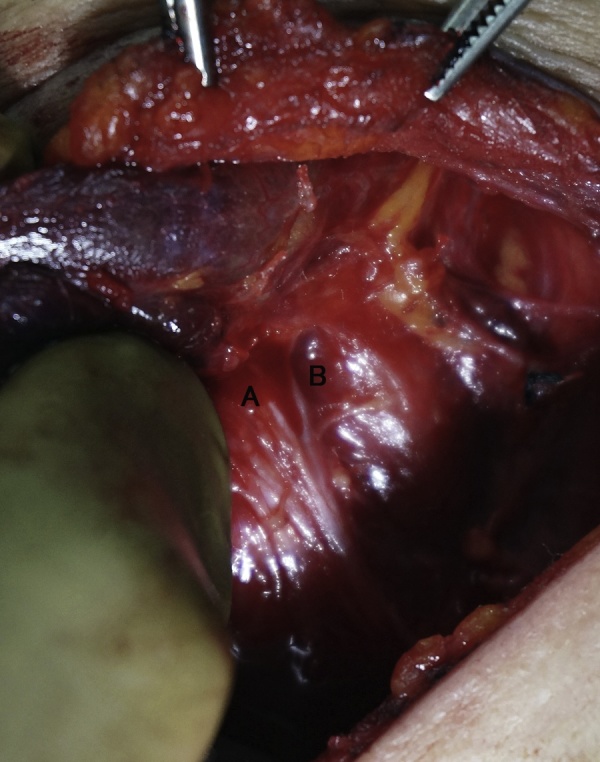
Identification and preservation of an extralaryngeal bifurcation of the recurrent laryngeal nerve by neuromonitoring during thyroid surgery in a 56-year-old woman. A: anterior branch, B: posterior branch.

We elected to perform surgery as per the patient’s desire for resection and a suspicion of malignancy. During surgery, we found the left recurrent laryngeal nerve was adhered to the tumor. We carefully detached the nerve from the tumor and traced it cranially. At this point, we observed a thin band that appeared to cross the nerve (Fig. 2). As a precautionary measure, we performed intraoperative neuromonitoring with an electromyography endotracheal tube (NIM-Response3.0, Medtronic Japan Co. Ltd.). A response was observed and identified to originate from an anterior extralaryngeal branch of the recurrent laryngeal nerve. We were able to preserve both the anterior and posterior branches, while safely resecting the left thyroid lobe.

Histopathological examination of the resected specimen revealed an adenomatous nodule measuring 4.7 × 3.2 × 3.1 cm in size.

The patient demonstrated favorable postoperative recovery and was discharged on day 7 with no hoarseness.

## 3 Discussion

Recurrent laryngeal nerve damage during thyroid surgery can considerably alert the patient’s quality of life. Accordingly to past studies, the incidence of recurrent laryngeal nerve palsy after thyroid surgery ranges from 1.0% to 13.3% [[1], [2], [3], [4], [5], [6], [7]]. Thomusch et al reported that the incidences of transient and permanent recurrent laryngeal nerve palsy were 2.1% and 1.1%, respectively [1]. Risk factors for recurrent laryngeal nerve palsy include reoperation [4], malignant tumors [5], and concomitant node dissection [7].The recurrent laryngeal nerve can demonstrate various anomalies and bifurcation, such as a nonrecurrent inferior laryngeal nerve associated with aberrant origin of the right subclavian artery [8]. For the prevention of injury to the recurrent laryngeal nerve in such situations, surgery must be based on abundant experience and performed more carefully than usual.

The recurrent laryngeal nerve typically divides into two or three branches after entering the larynx. When there are two branches, the anterior and posterior branches constitute the motor and sensory branches respectively. When there are three branches, in most cases, the anterior and middle branches are the motor branches, while the posterior branch is the sensory branch. In some cases, the recurrent laryngeal nerve exhibits an extralaryngeal branch, at a more central position than usual, before entering the larynx. This phenomenon is kown as extralaryngeal bifurcation of the recurrent laryngeal nerve [[9], [10], [11], [12]]. Failure to notice and correctly identify this bifurcation leads to recurrent laryngeal nerve injury. In particular injury to the anterior branch, which is the motor branch, has a particularly large effect [11,12].

An endotracheal tube with attached electromyography electrodes was recently developed for intraoperative neuromonitoring during thyroid surgery [13]. Initially, intraoperative neuromonitoring did not find widespread use because of reports stating that it did not help in lowering the risk of recurrent laryngeal nerve palsy, the potential for false-positive findings, and the fear that it would interfere with training in surgical techniques. In a previous study, the use of intraoperative neuromonitoring in patients undergoing thyroid surgery was shown to have no significant benefit with regard to the incidence of recurrent laryngeal nerve palsy [14]. However, intraoperative neuromonitoring is being increasingly recognized as a useful tool for searching and identifying the recurrent laryngeal nerve, particularly in cases of advanced thyroid cancer, reoperation, and large goiters [15]. Recently this procedure was also shown to be useful for identifying the external branch of the superior laryngeal nerve [16,17] and nonrecurrent laryngeal nerve [18]. In the present case, intraoperative neuromonitoring helped us in identifying and preserving an extralaryngeal bifurcation of the recurrent laryngeal nerve.

As mentioned above, intraoperative neuromonitoring is associated with a high false-positive rate of 10%–30% [13]. That is, it occasionally fails to show a response even when there is no nerve paralysis. The most common reason for a false positive finding is improper positioning of the electrodes. Therefore, it is important to use standardized techniques that confirm to guidelines for recurrent laryngeal nerve monitoring [19,20].

## 4 Conclusion

Intraoperative neuromonitoring has recently come to be recognized as a useful tool for identifying and preserving not only the recurrent laryngeal nerve and its aberrations but also the external branch of the superior laryngeal nerve in cases of difficult thyroid surgery. We were able to identify and safely preserve an extralaryngeal bifurcation of the recurrent laryngeal nerve by intraoperative neuromonitoring, in a case of thyroid adenoma. Going forward, intraoperative neuromonitoring may become a necessity for recurrent laryngeal nerve palsy risk management in case of thyroid surgery [21].

## Conflicts of interest

The authors declare that they have no competing interests.

## Funding

This research did not receive any specific grant from funding agencies in the public, commercial, or not-for-profit sectors.

## Ethical approval

Institutional review board approval was exempted because all data were collected from clinical records and imaging systems for routine preoperative planning.

## Consent

The patients and her family provided written informed consent for publication of this report.

## Author contribution

MY performed the surgery and wrote the manuscript. YS and YO performed the surgery. MY, TO, TF, MO, YO, EO, and HO contributed to critical revision of the manuscript. EO gave the final approval for the manuscript. All authors have read and approved the final manuscript.

## Registration of research studies

This paper reports just the record of patient treatment. This is not a paper about research work involving human participants.

## Guarantor

Masatsugu Yano.

## Provenance and peer review

Not commissioned, externally peer reviewed.
